# Effects of systemic hypoxia on human muscular adaptations to resistance exercise training

**DOI:** 10.14814/phy2.12033

**Published:** 2014-06-11

**Authors:** Michihiro Kon, Nao Ohiwa, Akiko Honda, Takeo Matsubayashi, Tatsuaki Ikeda, Takayuki Akimoto, Yasuhiro Suzuki, Yuichi Hirano, Aaron P. Russell

**Affiliations:** 1 Department of Sports Sciences, Japan Institute of Sports Sciences, 3‐15‐1 Nishigaoka, KitaTokyo, Japan; 2 Centre for Physical Activity and Nutrition Research, School of Exercise and Nutrition Sciences, Deakin University, Burwood, Victoria, Australia; 3 Division of Regenerative Medical Engineering, Center for Disease Biology and Integrative Medicine, Graduate School of Medicine, University of Tokyo, 7‐3‐1 Hongo, BunkyoTokyo, Japan

**Keywords:** Capillarization, resistance exercise training, skeletal muscle, systemic hypoxia

## Abstract

Hypoxia is an important modulator of endurance exercise‐induced oxidative adaptations in skeletal muscle. However, whether hypoxia affects resistance exercise‐induced muscle adaptations remains unknown. Here, we determined the effect of resistance exercise training under systemic hypoxia on muscular adaptations known to occur following both resistance and endurance exercise training, including muscle cross‐sectional area (CSA), one‐repetition maximum (1RM), muscular endurance, and makers of mitochondrial biogenesis and angiogenesis, such as peroxisome proliferator‐activated receptor‐*γ* coactivator‐1*α* (PGC‐1*α*), citrate synthase (CS) activity, nitric oxide synthase (NOS), vascular endothelial growth factor (VEGF), hypoxia‐inducible factor‐1 (HIF‐1), and capillary‐to‐fiber ratio. Sixteen healthy male subjects were randomly assigned to either a normoxic resistance training group (NRT,* n *=**7) or a hypoxic (14.4% oxygen) resistance training group (HRT,* n *=**9) and performed 8 weeks of resistance training. Blood and muscle biopsy samples were obtained before and after training. After training muscle CSA of the femoral region, 1RM for bench‐press and leg‐press, muscular endurance, and skeletal muscle VEGF protein levels significantly increased in both groups. The increase in muscular endurance was significantly higher in the HRT group. Plasma VEGF concentration and skeletal muscle capillary‐to‐fiber ratio were significantly higher in the HRT group than the NRT group following training. Our results suggest that, in addition to increases in muscle size and strength, HRT may also lead to increased muscular endurance and the promotion of angiogenesis in skeletal muscle.

## Abstract (precis)

This study investigated the effect of resistance exercise training performed under systemic hypoxia or normoxia on biochemical and molecular muscular adaptations in healthy male subjects. Our findings demonstrate that resistance training under systemic hypoxia led not only muscle hypertrophy, but most interestingly, to a greater increase in muscular endurance. This increase in muscular endurance was potentially caused by the increased angiogenesis as determined by capillary‐to‐fiber ratio.

## Introduction

Skeletal muscles undergo structural and functional adaptations to various stimuli including mechanical (e.g., exercise) and environmental (e.g., systemic hypoxia) stimuli. Endurance exercise training results in improved muscle oxidative capacity (Holloszy and Booth 1976), whereas resistance exercise training leads to increases in muscle size and strength (McDonagh and Davies 1984). Endurance exercise training (5–6 times/week for 3–6 weeks at 70–80% maximal oxygen uptake) performed in systemic hypoxia induces a greater increase in muscle oxidative capacity when compared to endurance exercise training under normoxia (Desplanches et al. 1993; Geiser et al. 2001). This suggests that skeletal muscle adaptations are specific to the type of exercise stimuli, and that the combination of exercise and systemic hypoxia may have a synergistic effect on skeletal muscle adaptations such as muscular endurance.

It is generally recognized that endurance exercise training causes a significant increase in skeletal muscle capillarization, characterized by an elevated capillary density and capillary‐to‐fiber ratio (Andersen 1975; Brodal et al. 1977; Hudlicka et al. 1992). This physiological adaptation contributes to enhanced aerobic capacity via an increase in the transport, conductance, and extraction of oxygen in skeletal muscle. Vascular endothelial growth factor (VEGF) (Folkman and Shing 1992; Hudlicka et al. 1992; van Weel et al. 2004; Olfert et al. 2009), the generation of nitric oxide by nitric oxide synthase (NOS) (Baum et al. 2004, 2013), and peroxisome proliferator‐activated receptor‐*γ* coactivator‐1*α* (PGC‐1*α*) (Arany et al. 2008; Leick et al. 2009) are positive regulators of angiogenesis in skeletal muscle. Among these regulators, VEGF is known to play a critical role in increasing angiogenesis. When compared with wild‐type mice, VEGF transgenic mice (van Weel et al. 2004) and muscle‐specific VEGF knock‐out mice (Olfert et al. 2009), respectively, have increased and decreased skeletal muscle capillary density. Both NOS (Baum et al. 2013) and PGC‐1*α* (Leick et al. 2009) knock‐out mice have decreased skeletal muscle VEGF expression and capillary‐to‐fiber ratio. Acute high‐intensity resistance exercise (three sets of 10 repetitions of two legged knee extensor exercise at 60–80% of 1RM) in humans increases the expression of skeletal muscle VEGF mRNA and protein (Gavin et al. 2007). Hypoxic stimuli in cells also increase VEGF mRNA levels through the activation of the nuclear transcription factor, hypoxia‐inducible factor‐1 (HIF‐1) (Forsythe et al. 1996). Thus, it is possible that resistance exercise training under systemic hypoxia, when compared with normoxia, causes a greater increase in skeletal muscle VEGF and capillarization potentially leading to increased muscular endurance.

Therefore, this study investigated the effects of resistance exercise training under systemic hypoxia on the angiogenic response and muscular endurance in human skeletal muscle. We hypothesized that resistance exercise training under systemic hypoxia would lead to a greater development of muscular endurance and greater increase in angiogenic and mitochondrial responses as demonstrated by increases in VEGF, PGC‐1*α*, NOS, and capillary‐to‐fiber ratio.

## Methods

### Subjects

Sixteen healthy, nonsmoking, male subjects participated in this study. The subjects were randomly assigned to either the normoxic resistance training group (NRT, *n *=**7) or the hypoxic resistance training group (HRT, *n *=**9). The physical characteristics of the subjects are shown in Table 1. All subjects performed exercise two to three times per week and had experience of recreational resistance training within the past 10 years. None of the subjects were involved in a regular resistance training program for at least 6 months prior to this study. Before or during participation in this study, subjects reported no exposure to an altitude of >3000 m within 1 month before the experimental trial, no history of severe acute mountain sickness, and no medications (e.g., anabolic steroids, creatine, or sympathoadrenal drugs) were taken during the experimental period. The experimental procedure as well as the purpose of this study was explained in detail to the subjects, and their written informed consent was obtained in advance. The study was approved by the Japan Institute of Sports Sciences Ethics Committee. This study was done from September to December in 2009.

**Table 1.** Physical characteristics of the subjects

Variable	Baseline	8 weeks
Age (year)		
NRT	28.2 ± 1.4	
HRT	28.4 ± 1.6	
Height (cm)		
NRT	170.2 ± 1.4	
HRT	171.1 ± 1.4	
Body mass (kg)		
NRT	65.8 ± 3.7	66.8 ± 3.7*
HRT	68.2 ± 2.2	69.4 ± 2.2*
Lean body mass (kg)		
NRT	53.8 ± 2.6	55.8 ± 3.0**
HRT	56.9 ± 1.7	58.2 ± 1.7**
% fat		
NRT	17.3 ± 1.8	16.4 ± 1.7*
HRT	16.1 ± 1.3	16.0 ± 1.4*

NRT, normoxic resistance training; HRT, hypoxic resistance training.

Values are represented as means ± SE.

Significantly different from baseline: *P <**0.05; **P <**0.01.

### Resistance exercise training protocol

The subjects performed 8‐week resistance training on nonconsecutive days (every Monday and Thursday or every Tuesday and Friday; 16 sessions in total). Except for the 1st and 16th training session, the HRT group was exposed to normobaric hypoxia conditions (14.4% oxygen) from 10 min before the resistance exercise session until 30 min after the exercise session. To investigate acute hormonal responses, the HRT group was exposed to hypoxic conditions from 15 min before the resistance exercise session until 60 min after the exercise session during the 1st and 16th training session. The resistance exercise consisted of two consecutive exercises (free‐weight bench‐press and bilateral leg‐press using weight‐stack machine), each with 10 repetitions for five sets at 70% of pretraining one‐repetition maximum (1RM). All the sets and exercises were separated by 90‐s rest intervals. The training intensity was increased (2.5 kg in bench‐press and 5.0 kg bilateral leg‐press, respectively) in subsequent training sessions when a subject completed 10 repetitions for five sets, or the training intensity was adjusted to 70% of each new 1RM after determination of 1RM after 2 weeks of training. The subjects were instructed to lift and lower the load at a constant velocity, taking ~2 sec (bench‐press) or 4 sec (bilateral leg‐press) for each repetition. The total exercise time was approximately 20 min at each session. If the load became too heavy, the subject was assisted. All training sessions were performed in the strength and conditioning facilities at the Japan Institute of Sports Sciences (Tokyo, Japan) under the supervision of assistant trainers. The HRT group used a specially designed hypoxic training room within the facility. The hypoxic environment was created by filtering compressed air through a high‐polymer membrane. The subjects were asked to refrain from performing other resistance training during the experiment period.

### Measurements of body composition

Body mass was measured with an electric scale to the nearest 0.1 kg before and after the training period. Lean body mass and percentage body fat were measured by dual‐energy X‐ray absorptiometry (DXA, QDR‐4500A, Hologic, Waltham, MA) before and after the training period. The subjects did not perform any strenuous exercise 48 h before measuring body composition.

### Measurements of muscle cross‐sectional area

The cross‐sectional area (CSA) of the right femoral region was measured using 1.5‐T magnetic resonance imaging (MRI) with a body coil (Magnetom Symphony, Siemens, Erlanden, Germany) before and after the training period. The CSA of the femoral muscle was calculated using T1‐weighted cross‐sectional images of the 50% area between the upper end of greater trochanter and the knee joint gap (spin echo method; repetition time: 404 ms; echo time: 11 ms, averaging parameter: 1; field of view: 240 × 240 mm; slice thickness: 10 mm; matrix: 256 × 256; imaging time: 3 min 34 sec). The muscle cross section was defined as the sum of the cross‐sectional areas of the *quadriceps femoris*,* biceps femoris*,* semitendinosus*,* semimembranosus*,* adductor longus*,* adductor magnus*,* gracilis*, and *sartorius* muscles. The CSA was calculated by tracing each area using the special image analysis software (independent system for the imaging services provided by Hitachi Medical, Tokyo, Japan). Bone, fat, nerves, and blood vessels were carefully excluded from the areas being analyzed. The measurements of the CSA were done blinded by the same expert.

### Measurements of muscular strength

Muscular strength was evaluated as 1RM for the bench‐press and bilateral leg‐press exercises under normoxia. Before measuring 1RM, a warm‐up with 10 repetitions at 50% of the perceived 1RM and stretching of the major muscle groups subjected to the exercises were performed. Three to five maximal trials separated by 3–5 min of rest were used to determine individual 1RM for each resistance exercise. Furthermore, 1RM testing was performed before and after the training period.

### Evaluations of muscular endurance

Bilateral leg‐press exercise using weight‐stack machine was used for evaluations of muscular endurance. Before the training period, the muscular endurance of the bilateral lower limbs was evaluated by the maximal number of repetitions at 70% of pretraining 1RM under normoxia. After the training period, the muscular endurance was evaluated at 70% of posttraining 1RM. Muscular exhaustion was defined as the moment when the weight ceased to move or the subjects failed to maintain the prescribed pace. Any repetition that lacked the full range of movement was not counted. Following the test, the performed exercise volume was calculated (load × repetition) and used as a measure of muscular endurance.

### Physical activity and nutritional controls

Subjects were instructed to continue their normal dietary and physical activity practices throughout the experiment. In addition, subjects were instructed not to perform any exercise aside from activities of daily living for 48 h prior to the biopsy procedures and exercise performance tests. Subjects were requested to consume their normal diet before and within 2 h after each training session.

### Blood sampling

#### Acute response to resistance exercise

Following an overnight fast, the subjects arrived at the training facility and rested for 30 min before the first blood collection at the 1st and 16th exercise training. The exercises were performed between 08:30 and 11:00 am. to avoid diurnal variations in the hormonal responses. Venous blood samples were obtained from each subject's forearm before normoxia and hypoxia exposures (pre 1), 15 min after normoxia and hypoxia exposures (pre 2), and at 0 (immediately after the exercises), 15, 30, and 60 min after exercise. Exposure to normoxia and hypoxia was continued until the experimental trials ended (60 min after exercises). Serum samples were separated from blood cells by centrifugation (1710 *g* for 10 min) and stored at −80°C until analysis.

#### Long‐term effects of resistance training

Resting blood samples were obtained from each subject's forearm under fasting conditions in the morning (between 08:00 and 09:00 am) at pretraining, week 4, and week 8 of the 8‐week intervention. Before blood sampling, subjects were asked to ensure a period of 48 h without any exercise activity. Subsequently, whole blood was collected and processed as previously described.

### Biochemical analysis

Serum GH concentration and insulin‐like growth factor 1 (IGF‐1) were measured by immunoradiometric assay (IRMA) using commercial kits (TFB, Tokyo, Japan and Mitsubishi Chemical Medience, Tokyo, Japan). The interassay and intraassay coefficients of variation (CVs) were 6.4% and 4.4% for GH and 6.9% and 4.0% for IGF‐1, respectively. Serum total testosterone, cortisol, and IGF binding protein‐3 (IGFBP‐3) levels were measured by radioimmunoassay (RIA) using commercial kits (DPC, Tokyo, Japan, TFB, Tokyo, Japan, ARC‐950; ALOKA, Tokyo, Japan). The interassay and intraassay CVs were 7.8% and 6.6% for testosterone, 6.2% and 5.0% for cortisol, and 10.2% and 8.6% for IGFBP‐3, respectively. The plasma VEGF concentration was measured by enzyme immunoassay (EIA) using a commercial kit (R&D systems, Minneapolis, MN). The interassay and intraassay CVs were 7.0% and 4.5% for VEGF, respectively. Red blood cell (RBC), hemoglobin (Hb), and hematocrit (Hct) values were obtained using an automated cell counter (SE‐9000; Sysmex, Hyogo, Japan). Arterial oxygen saturation (SpO_2_) was measured by pulse oximetry from the index finger (PULSOX‐Me300; TEIJIN, Tokyo, Japan).

### Muscle biopsy sampling

Skeletal muscle samples were obtained under local anesthesia (1% xylocaine) from the middle portion of the right *vastus lateralis* muscle using a percutaneous needle biopsy technique (Bergstrom 1975). A single incision was made in the skin and individual muscle samples were taken. The pretraining and posttraining biopsies were taken, respectively, 1 week before training and 48–96 h following the last training session. Muscle tissue was immediately frozen in liquid nitrogen and stored at −80°C until subsequent analysis.

### Reverse transcription

Total RNA was isolated from ~30 mg of skeletal muscle tissue with RNeasy Fibrous Tissue Mini Kit according to the manufacturer's protocol (Qiagen, Tokyo, Japan). The RNA concentration was determined spectrophotometrically at 260 nm (ES‐2; Malcom, Tokyo, Japan). Then, 1 *μ*g of total RNA was reverse transcribed into cDNA using a PrimeScript RT Reagent Kit (Takara, Siga, Japan), following the manufacturer's instructions. The reaction was performed at 37°C for 15 min. All isolated RNA and cDNA samples were stored −80°C until further analysis.

### Real‐time PCR analysis

Determination of relative mRNA expression was performed by real‐time PCR, using the Thermal Cycler Dice Real‐Time system (TP860, Takara). cDNA was diluted (1:5) and analyzed using SYBR Premix EX Taq II (Takara). The reaction vessel contained 12.5 *μ*L of SYBR Premix Ex Taq II, 1.0 *μ*L of forward and reverse primers, 2.0 *μ*L of cDNA, and a known amount of sterile water. The total volume of the reaction tube was 25 *μ*L. An initial cycle for 30 sec at 95°C was used to denature the cDNA. This was followed with 40 PCR cycles consisting of denaturation at 95°C for 5 sec, and primer annealing and extension at 60°C for 30 sec. The expression of glyceraldehyde‐3‐phosphate dehydrogenase (GAPDH) mRNA was determined as an internal control. The relative expression of target mRNA was normalized to the amount of GAPDH in the same cDNA by using the standard curve method described by the manufacturer. Dissociations curves verified that a single PCR product was amplified. Primer sequences are presented in Table 2.

**Table 2.** Primer sequences for real‐time PCR

Target gene	Sense primer	Antisense primer	GeneBank No.	Size (bp)
HIF‐1α	5′‐TCTGGGTTGAAACTCAAGCAACTG‐3′	5′‐CAACCGGTTTAAGGACACATTCTG‐3′	NM_001530.3	150
VEGF	5′‐GAGCCTTGCCTTGCTGCTCTAC‐3′	5′‐CACCAGGGTCTCGATTGGATG‐3′	NM_001025366.1	148
FLT‐1	5′‐GCGCTTCACCTGGACTGACA‐3′	5′‐GAAACTGGGCCTGCTGACATC‐3′	NM_002019.3	109
PGC‐1α	5′‐TTGATGCGCTGACAGATGGAG‐3′	5′‐TGTTGGCTGGTGCCAGTAAGAG‐3′	NM_013261.3	135
GAPDH	5′‐GCACCGTCAAGGCTGAGAAC‐3′	5′‐TGGTGAAGACGCCAGTGGA‐3′	NM_002046.3	138

HIF‐1α, hypoxia‐inducible factor 1 alpha; VEGF, vascular endothelial growth factor; FLT‐1, VEGF receptor 1; PGC‐1α, peroxisome proliferator‐activated receptor gamma coactivator 1 alpha; GAPDH, glyceraldehyde‐3‐phosphate dehydrogenase.

### Western blot analysis

Tissues were homogenized with 1x RIPA buffer (Millipore, Sydney, Australia) with 1 *μ*L/mL protease inhibitor cocktail (Sigma‐Aldrich, St. Louis, MO) and 10 *μ*L/mL Halt Phosphatase Inhibitor Single‐Use Cocktail (Thermo Scientific, Rockford, IL). The lysate was rotated overnight at 4°C before being centrifuged at 16,800 *g* at 4°C 5 min. Total protein concentration was identified using a BCA protein assay kit (Pierce Biotechnology, Rockford, IL), according to the manufacturer's instructions. Protein samples (60–100 *μ*g) were denatured at 95°C for 5 min in Laemmli buffer and separated by 8% or 15% SDS‐polyacrylamide gel and transferred to a polyvinylidene difluoride (PVDF, Millipore, Billerica, MA) membrane. Membranes were then incubated at room temperature for 1 h in blocking buffer supplemented with 5% bovine serum albumin (BSA) in phosphate‐buffered saline (PBS), after which they were incubated at 4°C with following primary antibodies diluted 1:1000 in 5% BSA in PBS (endothelial nitric oxide synthase (eNOS), BD Transduction Laboratories^™^, San Diego, CA (#610297); neuronal nitric oxide synthase (nNOS), BD Transduction Laboratories^™^ (#610309); vascular endothelial growth factor‐B (VEGF‐B), Santa Cruz Biotechnology, Dallas, TX (sc‐13083); Peroxisome proliferator‐activated receptor‐*γ* coactivator‐1*α* (PGC‐1*α*), Merck Millipore, Billerica, MA (#516557)). Following overnight incubation, the membranes were washed and incubated for 1 h at room temperature with infrared fluorescent dye‐conjugated secondary antibodies, either a goat anti‐mouse IgG (1:5000 [eNOS and nNOS] dilution, Alexa Fluor 680^®^, Invitrogen, Carlsbad, CA) or a goat anti‐rabbit IgG (1:5000 [PGC‐1*α*] or 1:25,000 [VEGF‐B] dilution, Alexa Fluor 800^®^, LI‐COR Biosciences, Lincoln, NE). After washing, proteins were exposed on an Odyssey^®^ Infrared Imaging System (LI‐COR Biosciences). GAPDH (Sigma‐Aldrich, St. Louis, MO [G8795]) was used to control for protein loading and individual protein band optical densities were determined using the Odyssey^®^ Infrared Imaging System software.

### Citrate synthase assay

Citrate synthase (CS) activity, a commonly used marker of mitochondrial mass and cellular oxidative capacity, was determined using a method described by Srere (1969), with minor modifications. Eight *μ*g of protein sample was added to 66 mmol/L Tris buffer, 0.1 mmol/L DTNB, 0.5 mmol/L Acetyl CoA and 0.6 mmol/L Oxaloacetate in 96‐well microplate at 25°C. Change in absorbance at 412 nmol/L was measured every 30 sec for 5 min using the BioTek Synergy 2 multi‐mode microplate reader (BioTek, Winooski, VT).

### Immunohistochemistry

Frozen muscle was placed in the optimum cutting temperature embedding medium (OCT; Tissue‐Tek) with its fibers perpendicular to the horizontal plane. Frozen sections (10 *μ*m) were cut using a cryostat and placed onto slides (Matsunami Glass Ind., Tokyo, Japan). Before being stained, the sections were fixed in 4% paraformaldehyde‐PBS for 5 min at 4°C, permeabilized with 0.3% Triton X‐100‐PBS for 10 min at 4°C, and then blocked in 5% normal goat serum (NGS)‐PBS for 30 min at room temperature, followed by incubation with monoclonal mouse anti‐human CD31 endothelial cell antibody (DAKO, Tokyo, Japan) diluted 1:100 in 5% NGS‐PBS at 4°C overnight. Two consecutive washes with PBS for 5 min each were followed by incubation with Alexa488‐conjugated donkey anti‐mouse IgG diluted 1:200 in 5% NGS‐PBS for 60 min at room temperature. After blocking with NGS‐PBS for 2 h at 4°C, incubation was performed with monoclonal mouse dystrophin antibody (Sigma) diluted 1:100 in 5% NGS‐PBS at 4°C overnight. Two consecutive washes with PBS for 5 min each were followed by incubation with Alexa549‐conjugated donkey anti‐mouse IgG diluted 1:100 in 5% NGS‐PBS for 60 min at room temperature. Images were captured under an Olympus confocal microscope. The numbers of CD31‐positive cells and muscle fibers in two fields of one cross‐section were manually counted with a Micro‐Analyzer (Nihon Poladigital, Tokyo, Japan). The capillary‐to‐fiber ratio was calculated by dividing the number of CD31‐positive cells by the number of muscle fibers, and the values were averaged.

### Statistical analysis

All data were analyzed by a two‐way ANOVA with repeated measures. When required a Fisher's PLSD *post hoc* analysis was performed. The level of statistical significance was set at *P *<**0.05. Data are expressed as mean ± SE.

## Results

### Body composition

In both the groups, body mass (*P *<**0.05) and lean body mass (*P *<**0.01) significantly increased, while percent body fat decreased after training (Table 1). There were no significant differences in these parameters between the two groups.

### Total training volume

Total training volume (calculated as load multiplied by repetitions) during the training period for the bench press was 36,500 ± 3933 kg for the NRT group and 42,000 ±3304 kg for the HRT group, and for bilateral leg‐press was 123,500 ± 10,795 kg for the NRT group and 129,111 ±6006 kg for the HRT group (n.s. between groups).

### Muscle cross‐sectional area and strength

**Figure 1.**
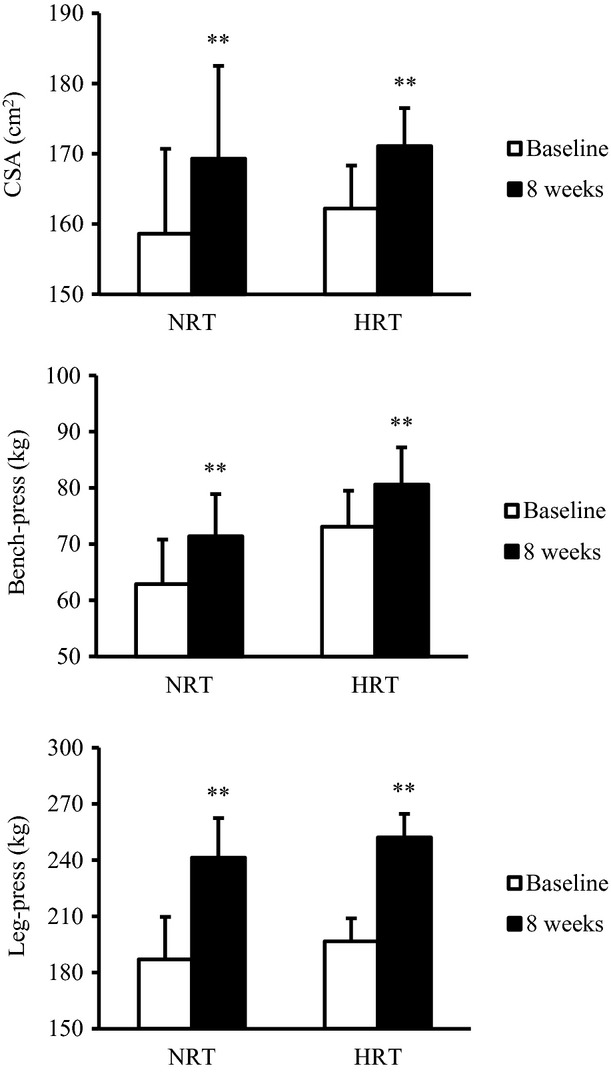
Changes in muscle cross‐sectional area (CSA) and one‐repetition maximum (1RM) of bench‐press and leg‐press before (baseline) and after (8 weeks) the resistance training program. NRT, normoxic resistance training (*n *=**7); HRT, hypoxic resistance training (*n *=**9). Significantly different from baseline: **P <**0.01.

In both groups, muscle CSA and 1RM significantly increased by 6.1% (CSA), 14% (1RM of bench‐press), and 31% (1RM of leg‐press) after training with no differences observed between groups (Figure 1) (*P *<**0.01).

### Muscular endurance

**Figure 2.**
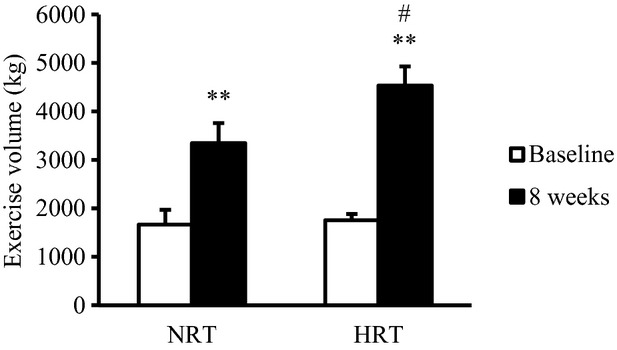
Changes in exercise volume during leg‐press exercises at 70% of 1RM before (baseline) and after (8 weeks) the resistance training program. NRT, normoxic resistance training (*n *=**7); HRT, hypoxic resistance training (*n *=**9). Values are represented as means ± SE. Significantly different from baseline: **P <**0.01; significantly different from NRT: ^#^*P *<**0.05.

Muscular endurance was evaluated as the exercise volume of bilateral leg‐press exercise performed at 70% 1RM (Figure 2). Muscular endurance significantly increased in both groups after training (*P *<**0.01). However, the exercise volumes were significantly higher in the HRT group than in the NRT group at the posttraining time point (*P *<**0.05) (Figure 2).

### Arterial oxygen saturation and acute GH responses

**Figure 3.**
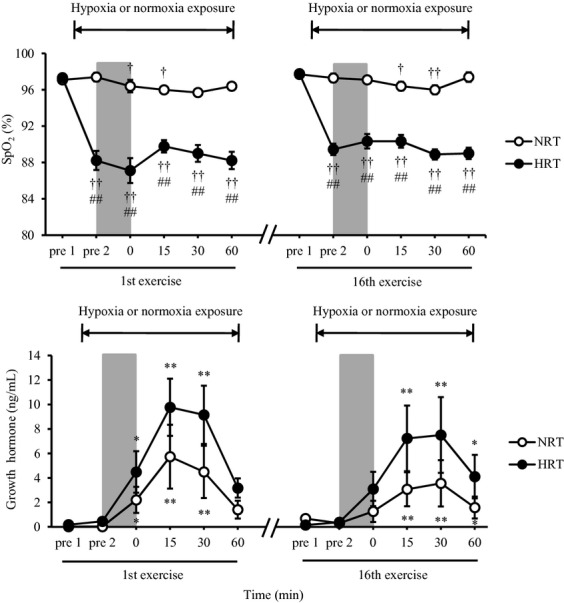
Changes in pulse oximetry oxygen saturation (SpO_2_) and growth hormone concentrations before and after the resistance exercises. NRT, normoxic resistance training (*n *=**7); HRT, hypoxic resistance training (*n *=**9). Values are represented as means ± SE. Significantly different from pre 1: ^†^*P *<**0.05; ^††^*P *<**0.01; significantly different from pre 2: *P <**0.05; **P <**0.01; significantly different from NRT: ^##^*P *<**0.01. Resistance exercise is denoted as a *shaded area*.

Figure 3 shows the SpO_2_ and GH concentrations measured before and after exercise. In the NRT group, SpO_2_ significantly decreased at 0 (1st exercise, *P *<**0.05), 15 (1st and 16th exercises, *P *<**0.05), and 30 (16th exercise, *P *<**0.01) min after exercise compared with pre 1 (before hypoxia exposure) value. In contrast, SpO_2_ in the HRT group was significantly lower at all‐time points after exposure to hypoxia (from pre 2 to 60 min after exercise) when compared to the pre 1 (*P *<**0.01). Moreover, SpO_2_ in the HRT group was lower than that in the NRT group at all‐time points after hypoxia exposure (*P *<**0.01). GH concentrations in both groups significantly increased after the 1st and 16th exercises (*P *<**0.05). In addition, GH concentration in the HRT group tended to be higher than that in the NRT group (ANOVA, *P *=**0.06).

### Resting levels of hormonal and hematological parameters

Resistance training decreased the cortisol concentration by 31% and increased T/C ratio by 73% independent of the training condition (Table 3) (*P *<**0.01). There was no significant difference in the cortisol concentration and T/C ratio between the two groups. Testosterone, IGF‐1, IGFBP‐3, RBC, HB, and Hct levels did not change throughout the training program in either training condition.

**Table 3.** Changes in testosterone, cortisol, testosterone/cortisol (T/C) ratio, insulin‐like growth factor‐1 (IGF‐1), IGF binging protein‐3 (IGFBP‐3), red blood cell (RBC), hemoglobin (Hb), and hematocrit (Hct) before (baseline), during (4 weeks), and after (8 weeks) the resistance training program

Variable	Baseline	4 weeks	8 weeks
Testosterone (ng/mL)			
NRT	5.74 ± 0.93	5.32 ± 0.64	4.54 ± 0.79
HRT	3.98 ± 0.45	5.20 ± 0.33	4.65 ± 0.22
Cortisol (μg/dL)			
NRT	14.4 ± 1.0	12.0 ± 1.5 **	9.3 ± 1.0 **
HRT	16.0 ± 1.7	11.4 ± 1.2 **	10.7 ± 1.0 **
T/C ratio			
NRT	4.03 ± 0.65	5.32 ± 1.27 **	5.64 ± 1.42 **
HRT	2.87 ± 0.61	5.13 ± 0.92 **	4.66 ± 0.46 **
IGF‐1 (ng/mL)			
NRT	234.4 ± 21.2	233.9 ± 22.3	232.4 ± 17.1
HRT	246.8 ± 24.9	261.1 ± 21.5	267.6 ± 21.9
IGFBP‐3 (ng/mL)			
NRT	2.56 ± 0.10	2.46 ± 0.19	2.47 ± 0.19
HRT	2.19 ± 0.17	2.55 ± 0.16	2.67 ± 0.14
RBC (/L)			
NRT	465.5 ± 51.1	498.4 ± 13.6	500.5 ± 8.1
HRT	495.0 ± 9.3	503.9 ± 8.6	494.2 ± 9.6
Hb (g/dL)			
NRT	15.4 ± 0.3	15.3 ± 0.3	15.3 ± 0.3
HRT	14.8 ± 0.2	15.3 ± 0.2	15.0 ± 0.3
Hct (%)			
NRT	47.7 ± 1.1	46.8 ± 1.0	46.5 ± 0.9
HRT	46.0 ± 0.6	46.4 ± 0.4	45.2 ± 0.7

NRT, normoxic resistance training; HRT, hypoxic resistance training.

Values are represented as means ± SE.

Significantly different from baseline: **P <**0.01.

**Figure 4.**
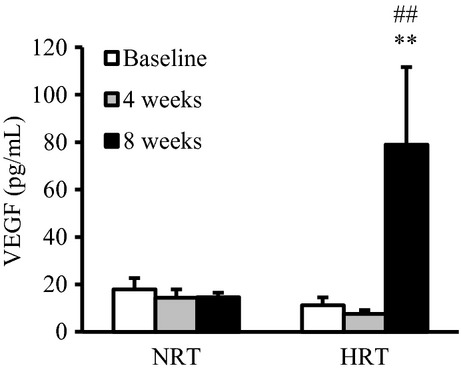
Changes in plasma vascular endothelial growth factor (VEGF) before (baseline), during (4 weeks), and after (8 weeks) the resistance training program. NRT, normoxic resistance training (*n *=**7); HRT, hypoxic resistance training (*n *=**9). Values are represented as means ± SE. Significantly different from baseline: **P <**0.01; significantly different from NRT: ^##^*P *<**0.01.

Circulating VEGF levels in the HRT group, but not be NRT, significantly increased after training (Figure 4) (*P *<**0.01).

### mRNA expressions in skeletal muscle

**Figure 5.**
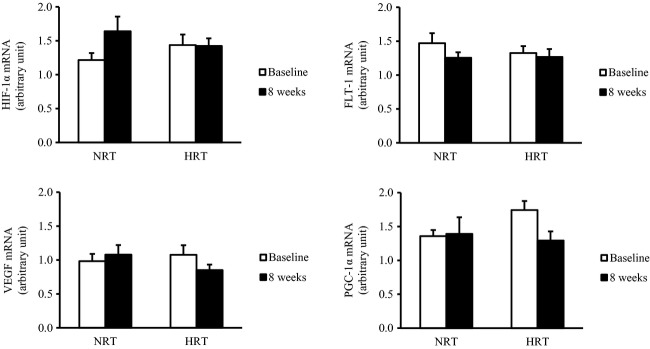
Hypoxia‐inducible factor 1 alpha (HIF‐1*α*), vascular endothelial growth factor (VEGF), VEGF receptor 1 (FLT‐1), and peroxisome proliferator‐activated receptor‐*γ* coactivator‐1*α* (PGC‐1*α*) mRNA expressions in skeletal muscle before (baseline) and after (8 weeks) the resistance training program. NRT, normoxic resistance training (*n *=**7); HRT, hypoxic resistance training (*n *=**9). Values are represented as means ± SE.

Training did not change the basal mRNA expressions levels of HIF‐1*α*, VEGF, FLT‐1, and PGC‐1*α* in skeletal muscle (Figure 5).

### Protein expressions in skeletal muscle

**Figure 6.**
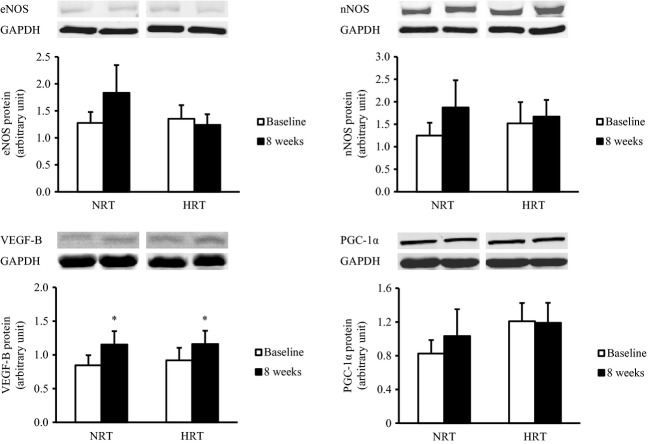
Endothelial nitric oxide synthase (eNOS), neuronal nitric oxide synthase (nNOS), vascular endothelial growth factor‐B (VEGF‐B), and peroxisome proliferator‐activated receptor‐*γ* coactivator‐1*α* (PGC‐1*α*) protein expressions in skeletal muscle before (baseline) and after (8 weeks) the resistance training program. NRT, normoxic resistance training (*n *=**7); HRT, hypoxic resistance training (*n *=**9). Values are represented as means ± SE.

Skeletal muscle VEGF‐B protein levels significantly increased by 51% after resistance training in both the groups (*P *<**0.05) (Figure 6). There was no effect of training of skeletal muscle eNOS, nNOS, and PGC‐1*α* protein levels (Figure 6).

### Citrate synthase assay

**Figure 7.**
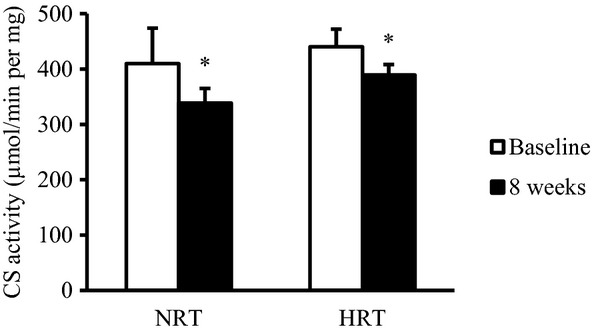
Change in citrate synthase (CS) activity before (baseline) and after (8 weeks) the resistance training program. NRT, normoxic resistance training (*n *=**7); HRT, hypoxic resistance training (*n *=**9). Values are represented as means ± SE. Significantly different from baseline: *P <**0.05.

In both the groups, CS activity significantly decreased by 9% after training (Figure 7) (*P *<**0.05), with no differences observed between groups.

### Capillarization

**Figure 8.**
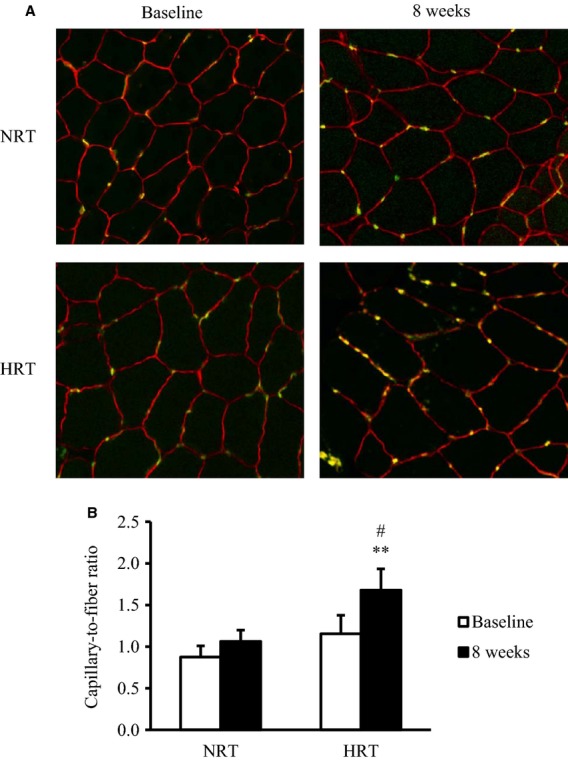
(A) Immunofluorescence staining of endothelial cells with anti‐CD31 antibody and fluorescein‐conjugated secondary antibody (green) in skeletal muscle before (baseline) and after (8 weeks) the resistance training program. (B) Capillary‐to‐fiber ratio before (baseline) and after (8 weeks) training program. NRT, normoxic resistance training (*n *=**6); HRT, hypoxic resistance training (*n *=**6). Values are represented as means ± SE. Significantly different from baseline: **P <**0.01; significantly different from NRT: ^#^*P *<**0.05.

Capillary‐to‐fiber ratio significantly increased by 52% in the HRT group after resistance training (Figure 8) (*P *<**0.01); a trend was observed for an increase in the NRT group (*P *=**0.06). As a result the capillary‐to‐fiber ratio was significantly higher in the HRT group than in the NRT group at the posttraining (*P *<**0.05).

## Discussion

Systemic hypoxia is a potent stimulator of endurance exercise adaptations. However, its effect of resistance training adaptations has received little attention. This study therefore investigated the effects of 8 weeks of resistance exercise training, performed under hypoxic (HRT) and normoxic conditions (NRT), on skeletal muscle adaptations known to occur following both resistance and endurance exercise training. Novel findings from this study included: (1) a significant improvement in muscular endurance; (2) increases in plasma VEGF concentration and capillary‐to‐fiber ratio following training under hypoxic conditions; and (3) similar gains in muscle CSA and strength, independent of the training protocol. These results demonstrate that resistance training under systemic hypoxia not only stimulates classical resistance training adaptations but also promotes adaptations more commonly associated with endurance exercise training in skeletal muscle.

For the first time, we demonstrate a significant resistance training‐induced increase in muscular endurance in the HRT group. Recently, Faiss et al. (2013) showed a larger improvement in repeated sprint performance following sprint training in hypoxia when compared to sprint training in normoxia. This was associated with significant increases in carbonic anhydrase III and monocarboxylate transporter 4 mRNA levels; a protein involved in muscle buffering capacity. Improvements in muscular endurance are influenced by increases in skeletal muscle oxidative fiber types, activities of metabolic enzymes, improvement in muscle buffering capacity, and capillarization. Our observed increase in muscular endurance following resistance exercise training under systemic hypoxia was paralleled by increases in the capillary‐to‐fiber ratio. As far as we aware no studies have examined the effect of resistance training under systemic hypoxia on muscle capillarization. However, it has been observed that endurance training under systemic hypoxia induces a greater increase in muscle capillary density and capillary‐to‐fiber ratio, respectively, than normoxia (Desplanches et al. 1993; Geiser et al. 2001). In addition, Vogt et al. (2001) reported greater increases in VEGF mRNA expression and capillary density in skeletal muscle by endurance training under systemic hypoxia. Combined these observations demonstrate that exercise training under systemic hypoxia may increase skeletal muscle angiogenesis, independent of the training mode. The greater angiogenesis is likely to be achieved by hypoxic resistance training‐induced increase in VEGF levels. We were not able to define whether the greater increase in muscular endurance in the HRT group was due to the promotion of angiogenesis in skeletal muscle and this should be the focus of future studies.

To our knowledge, this study is the first study to identify an increase in basal VEGF protein levels in skeletal muscle following resistance training. Basal VEGF mRNA levels do not change following high‐intensity resistance exercise training (Lundberg et al. 2013) or low‐intensity resistance training under systemic hypoxia (Friedmann et al. 2003). In addition, the basal VEGF mRNA levels in skeletal muscle did not change after high‐intensity intermittent training under systemic hypoxia. Following training in normoxia there are conflicting results with studies showing that VEGF mRNA levels may increase (Mounier et al. 2009) or decrease (Faiss et al. 2013). However, VEGF mRNA levels are increased between 2 and 4 h following moderate–high‐intensity acute resistance exercise (Gavin et al. 2007) and 4 and 24 h following low‐intensity acute resistance exercise performed with blood flow restriction‐induced local hypoxia (Larkin et al. 2012). The transient increase in VEGF mRNA following an acute resistance exercise bout may contribute to an increase in VEGF protein levels following repeated exercise bouts as observed in this study.

Some previous studies have obtained similar results regarding the changes of oxidative capacity to resistance training (MacDougall et al. 1979; Chilibeck et al. 1999; Masuda et al. 1999). Protein level of PGC‐1*α*, a key regulator of mitochondrial biogenesis, angiogenesis, and endurance capacity, did not change, while CS enzyme activity, another regulator of endurance capacity, and mitochondrial content were reduced, following training. Resistance training might induce muscular hypertrophy to a greater extent than mitochondrial biogenesis, resulting in decreased CS activity relative to the muscle fiber area. It is possible that the increase in endurance capacity following HRT was not significantly influenced by changes in mitochondrial biogenesis or oxidative capacity.

In this study, further gains in muscle size and strength after resistance training under systemic hypoxia were not observed. We have previously confirmed that the same resistance exercise protocol performed under hypoxia induces a greater response in GH which has anabolic effects as compared to resistance exercise under normoxia (Kon et al. 2010). However, a consensus view on the relationship between the change in GH following a single session of resistance exercise and muscular hypertrophy with resistance training has not been achieved so far (McCall et al. 1999; Mitchell et al. 2013). Additionally, GH administration does not have an additive effect muscle hypertrophy and strength when combined with resistance training in healthy elderly men (Lange et al. 2002). These observations suggest that the increase in GH following resistance exercise may not be implicated in the muscle hypertrophy and strength gain induced by such training.

The increase in T/C ratio in this study was caused by the resistance training‐induced decrease in cortisol and was independent of the training protocol. Testosterone is an androgenic‐anabolic hormone and plays a role in promoting muscle growth (Vingren et al. 2010). On the other hand, cortisol has catabolic functions that have greater effects in fast‐twitch muscle fibers (Crowley and Matt 1996). Therefore, T/C ratio has been suggested to be an indicator of the anabolic/catabolic status of skeletal muscle during resistance training (Häkkinen 1989). The present result suggests that systemic hypoxia does not affect the response of the resting T/C ratio to resistance training.

Several limitations need to be addressed. First, we could not use a crossover and blinded experiment design. Therefore, the performance of the muscular endurance test might be affected slightly by psychological parameters. As there was relatively large intersubjects variability in responses to hypoxia, studies using a crossover and blinded experiment design are required to reduce the intersubjects variability. Second, we could not investigate myofiber CSA by staining frozen *vastus lateralis* muscle sections although the muscle CSA was measured using MRI in this study. Therefore, we could not reveal if the increase of the muscle CSA was due to an increase in myofiber CSA or to an increase of the number of myofibers. Finally, the number of CD31‐positive cells and muscle fibers were measured in only two fields of view in this study. Future studies using more fields of view are required to investigate the effect of hypoxic resistance training on skeletal muscle angiogenesis in more detail.

## Conclusions

We demonstrated that resistance training under systemic hypoxia led to greater increases in muscular endurance and angiogenesis in the skeletal muscle.

## Acknowledgments

We thank members of the hypoxic research project in Japan Institute of Sports Sciences for their critical comments and excellent technical support. We also thank medical doctors, nurses, and clinical laboratory technicians in Japan Institute of Sports Sciences for help with the conduct of the clinical portion of this study. We thank Akisa Tobimatsu and Marita A. Wallace for their excellent technical assistances.

## Conflict of Interest

None declared.
